# Bioactive substances and potentiality of marine microalgae

**DOI:** 10.1002/fsn3.2471

**Published:** 2021-07-26

**Authors:** Jinhong Wu, Xinzhe Gu, Danlu Yang, Shannan Xu, Shaoyun Wang, Xu Chen, Zhengwu Wang

**Affiliations:** ^1^ South China Sea Fisheries Research Institute Chinese Academy of Fishery Sciences/Key Laboratory of South China Sea Fishery Resources Exploitation & Utilization Ministry of Agriculture and Rural Affairs/Guangdong Provincial Key Laboratory of Fishery Ecology and Environment Guangzhou China; ^2^ Department of Food Science and Engineering School of Agriculture and Biology Shanghai Jiao Tong University Shanghai China; ^3^ Scientific Observation and Research Field Station of Pearl River Estuary Ecosystem Guangzhou China; ^4^ College of Biological Science and Technology Fuzhou University Fuzhou China; ^5^ Southern Marine Science and Engineering Guangdong Laboratory Guangzhou China

**Keywords:** bioactive substances, health, microalgae, nutrition, potential application

## Abstract

Microalgae is one of the most important components in the aquatic ecosystem, and they are increasingly used in food and medicine production for human consumption due to their rapid growth cycle and survival ability in the harsh environment. Now, the exploration of microalgae has been gradually deepening, mainly focused on the field of nutrition, medicine, and cosmetics. A great deal of studies has shown that microalgae have a variety of functions in regulating the body health and preventing disease, such as nitrogen fixation, antitumor, antivirus, antioxidation, anti‐inflammatory, and antithrombotic. Furthermore, microalgae can synthesize various high‐valued bioactive substances, such as proteins, lipids, polysaccharides, and pigments. In this paper, we have briefly reviewed the research progress of main bioactive components in microalgae, proteins, lipids, polysaccharides, pigments, and other nutrients included, as well as their present application situation. This paper can provide the guidance for research and development of industrial production of microalgae.

## Graphical abstract

The manuscript has reviewed the research progress of main bioactive components in microalgae, proteins, lipids, polysaccharides, pigments, and other nutrients, as well as their potential application in industrial production. Simultaneously, this review has briefly demonstrated that microalgae have huge advantages in regulating the body health and preventing disease, such as nitrogen fixation, antitumor, antivirus, anti‐oxidation, anti‐inflammatory, and anti‐thrombotic.

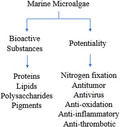

## 1 INTRODUCTION

Marine algae are divided into two types based on their form: microalgae, such as diatoms, dinoflagellates, and Cyanophyta, and the other is macroalgae, such as green, brown, or red algae. Microalgae represent the microscopic photosynthetic group that consists of eukaryotic organisms and prokaryotic cyanobacteria (Yan et al., 2016). Microalgae have vast biodiversity, but nearly unused resources. There are approximately 200,000–800,000 species predicted within numerous genera, including 50,000 that have been reported. They play a vital role on earth and forms the world's largest biomass, responsible for at least 32% of the world's photosynthesis (Priyadarshani et al., 2011).

Microalgae has a long history of consumption for more than 2,000 years in China. Due to the valuable microalgal metabolites, including pigments, polysaccharides, or polyunsaturated fatty acids (PUFAs), they have applications in many industries, food, health products, animal husbandry, aquatic feed, and cosmetics. Furthermore, there is growing interest in biomacromolecules as a potential drug, marine organisms being a great source. Hence, microalgae could be included in screening programs for promising bioactive substances.

Microalgae have much higher photosynthetic efficiency and are not sensitive to the seasons compared with higher plants, which allows for high yields of valuable molecules such as proteins, lipids, carbohydrates, and pigments, combined with the diversity of microalgae (Tarento et al., 2018; Welladsen et al., 2014; Yan et al., 2016). Next, microalgae can grow under less demanding conditions, such as wastewater or nonagricultural land. Moreover, microalgae can minimize associated environmental impacts by recycling atmospheric carbon dioxide.

In terms of food, resource, and environmental challenges, microalgae have a wide range of applications. At present, microalgae biotechnology is steadily rising, divided into four main research areas, including wastewater treatment, carbon dioxide sequestration, biofuel production, and high value‐added active substance production. The first three areas have been well explored and reviewed over the past few decades, and now a recent shift occurs in the focus of microalgae applications, where producers focus primarily on the production of high value‐added components rather than environmental applications. High value‐added ingredients from microalgae had a very broad category, ranging from lipids, proteins, and carbohydrates with food and nutritional applications to dyes and sterols with cosmetic and pharmaceutical applications. Hence, the core content of this paper summarizes the production, application, and potentiality of high value‐added active substances and points out the direction for future producers.

Moreover, marine microalgae are not only an essential part of the marine biological resources but also a major generator within the marine ecosystem. They play a crucial role in the material cycle and energy flow of the marine ecosystem and affect the productivity of the entire ocean system in direct or indirect ways, closely related to aquaculture, fishery resources, and geological and environmental protection.

## 2 PROTEINS

Marine microalgae have attracted attention because of its abundance, relatively inexpensive process, and a rich source of proteins and peptides with biological function (Rashidi et al., 2019; Zhang et al., 2019). Marine microalgae contain many active proteins with physiological functions. The main functions of these active substances include diazotrophic, antitumor, antioxidant, and immune‐stimulating activities. They can be used as components in medicinal cosmetics, and their bodies can be used as effective adsorbents for heavy metals in the ocean (Cornish & Garbary, 2010; Lee et al., 2013).

Generally, the main components in microalgae constitute of carbohydrates, proteins, and lipids. Due to the differences among microalgal species and cultivation conditions, the proportions of cellular components (carbohydrate, lipid, and protein) in microalgae can differ greatly. Marine microalgae are high in protein content, that is, 6%–70% of its dry weight (DW), and a majority having about 50%, as shown in Table 1. High protein content gives microalgae a high nutritional value. Thus, microalgal proteins can be used as a supplementary source of dietary protein, especially in some developing countries. Furthermore, the protein could be used as ingredients in healthy foods and even functional foods. With the continuous advancement in technology, active proteins from microalgae showed great potential and have become one of the research focuses.

**TABLE 1:** Percentage of proteins of different microalgae strains

Alga	Protein (%)	References
*Acutodesmus dimorphus*	28.1	Tibbetts et al. (2015)
*Anabaena cylindrica*	43–56	Becker (2007)
*Aphanizomenon flos‐aquae*	62	Becker (2007)
*Arthrospira maxima*	60–71	Becker (2007)
*Botryococcus braunii*	39	Tibbetts et al. (2015)
*Chlamydomonas rheinhardii*	48	Becker (2007)
*Chlorella pyrenoidosa*	57	Hamed et al. (2016)
*Chlorella vulgaris*	31.17	Phusunti et al. (2018)
*Chlorella sorokiniana*	18.81	Chen et al. (2014)
*Dunaliella salina*	57	Becker (2007)
*Dunaliella tertiolecta*	20–28	Welladsen et al. (2014)
*Entomoneis punctulata*	15–25	Welladsen et al. (2014)
*Neochloris oleoabundans*	30.1	Rashidi et al. (2019)
*Porphyridium cruentum*	28–39	Becker (2007)
*Scenedesmus almeriensis*	42.8	Vizcaíno et al. (2019)
*Scenedesmus obliquus*	50–56	Becker (2007)
*Spirulina platensis*	46–63	Tibbetts et al. (2015)
*Nannochloropsis oceanica*	52.63	Zhang et al. (2019)
*Nannochloropsis gaditana*	44.9	Vizcaíno et al. (2019)

### 2.1 Phycobiliproteins

Phycobiliproteins, the water‐soluble pigment–protein complex, exist in *Cyanobacteria* as well as some algae. The complex is formed based on apoprotein and covalently bound phycobilins serving as the chromophores. Phycobiliproteins are classified into four groups: phycocyanin (PC), phycoerythrin (PE), allophycocyanin (APC), and phycoerythrocyanin (PEC). PC was isolated from *Spirulina* and mainly exists in Cyanophyta, Rhodophyta, and Cryptophyta [80]. It is usually divided into C‐phycocyanin (C‐PC) and R‐phycocyanin (R‐PC), where the former is found in Cyanophyta, the latter in Rhodophyta, and both in Cryptophyta. PC is a protein–pigment complex that comprises of protein and nonprotein components, which participate in various biological effects, such as antioxidant, anti‐free radical, and antitumor activities (Eriksen, 2008). PC can also prevent oxidative stress (OS) through scavenging reactive oxygen species (ROS) as well as reactive nitrogen species (RNS). Romay, Armesto et al. (1998) were the first to report that PC possessed antioxidant property and also confirmed that it could scavenge superoxide anion and hydroxyl, alkoxyl radical. Besides, the effect of PC on inflammation was first described by these authors (Romay, Ledón et al., 1998). In addition, Liu et al. (2015) investigated the recovery effects of PC on the oxidative damage in mice caused by X‐ray radiation, and results proved that PC could effectively protect the body's antioxidant system and improve its antioxidant capacity. Many diseases have resulted from oxidative damage to the human body. PC could thus act as a nutraceutical in the clinical practice. Also, phycocyanin, as a natural blue pigment, has been widely utilized in cosmetics, such as lipstick, eyeliner, nail polish, or eye shadow (Jahan et al., 2017).

C‐PC, a natural blue pigment, is frequently found in *Cyanobacteria*, and R‐PC is isolated from red algae (Glazer, 1989). C‐PC exhibits an antitumor effect. C‐PC from *Oscillatoria tenuis* showed antioxidation and antiproliferation activity on human tumor cells *via* inducing cell apoptosis, including the representative apoptotic characteristics, such as cell contraction, membrane blebbing, condensed nucleus, or DNA fragmentation (Thangam et al., 2013). In addition, C‐PC suppressed HepG2 (human hepatoma cells) growth and multiplication at 7.0 µg/ml with an LC50 under 1.75 µg/ml (Basha et al., 2008). Cyclooxygenase 2 (COX‐2) is one of the induced enzymes with high expression in inflammatory and cancer cells. COX‐2 has been recently discovered to be related to colorectal cancer (CRC), breast cancer (BC), and gastric cancer (GC) (Cheng & Fan, 2013; Auberdiac et al., 2011; Liu et al., 2017). Saini and Sanyal (2014) suggested that C‐PC, an inhibitor of COX‐2, could reduce the tumor/lesion volume and number in the DMH‐induced CRC rat model. Jiang et al. found that C‐PC suppressed BC MDA‐MB‐231 cell proliferation through the activation of c‐Jun N‐terminal kinase (JNK) as well as p38 mitogen‐activated protein kinase (MAPK) signal transduction pathways and suppressing the extracellular signal‐regulated kinase (ERK) signal transduction pathway. It decreased the COX‐2 protein‐level while inhibiting MDA‐MB‐231 cell migration (Jiang et al., 2018).

R‐PC, a member of the phycobiliprotein family, has relatively limited studies. Chang et al. (2011) assessed the R‐PC treatment of allergic airway inflammation and reported elevated IL‐12 p70 levels in the monocyte‐derived dendritic cells in both healthy humans and patients with asthma. Moreover, Liu et al. (2015) determined that R‐PC had antiallergy potential after an experiment on mice or mast cells sensitized by antigens. It was also found that R‐PC effectively reduced tropomyosin and histamine levels and lowered interleukin 4 (IL‐4) and IL‐13 levels in mice. In addition, this protein also inhibited pro‐inflammatory factors and allergy markers, for example, suppressing the production of IL‐4 together with tumor necrosis factor‐α (TNF‐α), decreasing the histamine, β‐hexosaminidase, or ROS release in cells.

### 2.2 Collagen‐like protein

Collagen accounts for the major structural protein in the space outside cells of different animal connective tissues that showed extensive applications ranging from foods to medicines. It can also be adopted for burn or cosmetic operations (Jérome et al., 2015). There are different members in the collagen protein family, besides many novel proteins are under development by identifying the collagen‐like molecules such as TrpA. Recently, TrpA has been discovered and examined in a marine cyanobacterium, *Trichodesmium erythraeum* IMS 101. The phylogenetic analysis showed that the protein sequence of TrpA showed a high relationship with collagen proteins, not forming fibril. Scanning electron microscopy (SEM) results showed the expression of TrpA on trichome surface, with no typical localization pattern (Price et al., 2014). It has been reported that the TrpA gene showed expression at various growth stages, indicating its necessity for *T. erythraeum* growth. TrpA protein plays an important role in maintaining trichome structural integrity by adhering to cells nearby (Price & Anandan, 2013). *T*. *erythraeum* belonging to Cyanophyta, often proliferate to form red tide algae in autumn and winter. The presence of such a collagen gene with abnormal length in prokaryotes is possibly related to the capability of *T. erythraeum* to form blooms covering the ocean extensively (Layton et al., 2008). The collagen‐like protein from marine microalgae could thus be potentially used for cosmetic and medical formulation.

### 2.3 Diazotrophic protein

Some primary groups among nitrogen (N_2_) fixation microorganisms were studied. The N_2_ fixation microorganisms were less diverse based on nitrogenase gene (nifH) diversity. Most nifH sequences retrieved can be divided into two categories, including one constituted by *Trichodesmium* sequences and other by the *α‐proteobacterial* group. At present, *Trichodesmium* is the highest abundant diazotroph, with as high as 6 × 10^5^ nifH gene copies/L (Moisander et al., 2008). N_2_ fixation is catalyzed *via* the oxygen‐sensitive nitrogenase. However, there is no study on the mechanism of the nitrogenase from *Trichodesmium*.

*Cyanobacteria* represent the only diazotrophic microorganism producing O_2_ as the photosynthetic by‐product. Similar to eukaryotic algae and higher plants, they have chlorophyll, photolyze water, and release O_2_. It has been reported that one N_2_ fixation cyanobacteria, *Cyanothece* 51142, contained the highest complete adjacent N_2_ fixation‐associated gene cluster (Welsh et al., 2008). In 1970, soluble nitrogenase was first observed in vegetative cells of the blue‐green alga, *Anabaena cylindrica* (Smith & Evans, 1970). In 1979, the nitrogenase complex was originally separated by N‐starving *Anabaena cylindrica* culture. The Mo‐Fe protein having subunit composition, molecular weight (220,000 Da), isoelectric point (4.8), Mo (2 mol/mol), S^2−^ (20 mol/mol), and Fe (20 mol/mol) contents, as well as amino acid (AA) composition similar to components that contained Mo and Fe, was subjected to homogeneous purification and separated based on additional bacteria (Hallenbeck et al., 1979). Furthermore, diazotrophic *cyanobacteria* contribute to human health in two main ways. On the one hand, diazotrophic cyanobacteria enhance crop productivity in an environmentally friendly and economically viable manner. On the other hand, diazotrophic cyanobacteria are widely used as biofertilizer and soil conditioner to improve grain output, plant growth, fruit quality, and nutritional characteristics.

### 2.4 Bioactive peptides

Bioactive peptides (BP) as a specific protein fragment play a vital role in the physiological activity of most living organisms. BP was reported to have several therapeutic activities, including antioxidant, anti‐inflammatory, antitumoral, antiproliferative, antihypertensive, and antimicrobial properties, as shown in Table 2. Peptides derived from microalgae are known to show high potential in the fields of functional food, medicine, and cosmetics on account of their selectivity, efficacy, safety, and good tolerance once consumed. Furthermore, the interest in the microalgae‐derived bioactive peptides is increasing rapidly. Chen, Liou et al. (2011), Chen, Anaya et al. (2011) investigated the protective effects of peptide obtained from *Chlorella* on inhibiting MMP‐1 production induced by UVB and procollagen gene degradation in human skin fibroblasts. Shih and Cherng (2012) also attempted to explain the mechanism of a peptide derived from *Chlorella* against the cytotoxicity induced by UVB. The results showed that *Chlorella‐*derived peptide could inhibit cytotoxicity caused by UVC, reduce pFADD expression, decrease cleaved PARP‐1, and prevent DNA damage and fragmentation. In addition, De Lucia et al. isolated spirulina*‐*derived peptides as a novel biotechnological active ingredient that could increase hydration and reduce osmotic stress in skin cells. Sedighi et al. (2019) evaluated the antibacterial activity of peptide fractions of *Chlorella vulgaris*, which were digested by pepsin and had antimicrobial activity against *E. coli* CECT 434. Guzmán et al. (2019) reported antibacterial peptides from *Tetraselmis suecica*, namely AQ‐1766 peptide (LWFYTMWH), which had antimicrobial activity against the Gram‐negative bacteria, such as *E. coli*, *P. aeruginosa* and *S. typhimurium*, as well as against the Gram‐positive bacterial strains, for example, *B. cereus*, *M. luteus*, *L. monocytogenes*, and methicillin‐resistant *S. aureus*. Several studies have also demonstrated that peptides from microalgae had antitumoral and antihypertensive activity (Table 2).

**TABLE 2:** The biological activity of bioactive peptides from different strains

Activity	Bioactive peptides	References
Antioxidant activity	Tetrapeptide MGRY (MW = 526) from *Pavlova lutheri* showed the ability to scavenge for free radicals	Oh et al. (2015)
Antiproliferative activity	Peptide obtained from *Chlorella* inhibited UVB‐induced c‐fos and c‐jun expressions	Chen et al. (2011)
	Peptide derived from chlorella inhibited cytotoxicity caused by UVC, reduce pFADD expression, decrease cleaved PARP‐1, and prevent DNA damage and fragmentation	Fen and Yuh (2012)
Anti‐inflammatory activity	Spirulina*‐*derived peptides increased hydration and reduce osmotic stress in skin cells	Chiu‐Lan et al. (2011)
Antimicrobial activity	Protein fraction of *Chlorella vulgaris* with 62 kDa had antimicrobial activity against *E. coli* CECT 434	Sedighi et al. (2019)
	AQ‐1766 peptide (LWFYTMWH) from *Tetraselmis suecica* had antimicrobial activity against the Gram‐negative and Gram‐positive bacterial strains	Guzmán et al. (2019)
Antitumoral activity	Suppress PMA‐induced secretion of matrix metalloproteinase‐9 through the inactivation of the JNK, p38, and NF‐κB pathways in human fibrosarcoma cells	Heo et al. (2018)
Antihypertensive activity	Peptide fractions isolated from *Spirulinas* inhibited angiotensin I converting enzyme	Heo et al. (2017)
	Peptides from *Vischeria helvetica KGU‐Y001* had molecular weights lower than 400 Da and high ACE inhibitory activities	Aburai et al. (2020)
	Peptides from *Chlorella sorokiniana* showed ACE inhibitory activity	Lin et al. (2018)

## 3 LIPIDS

Microalgal oil has attracted much attention due to its short breeding cycle, its ability to capture carbon dioxide and accumulate a large amount of lipids, and its rich omega‐3 long‐chain polyunsaturated fatty acids. As shown in Table 3, the fatty acids of microalgae oil mainly include saturated fatty acids, monounsaturated fatty acids, and polyunsaturated fatty acids. Usually, polyunsaturated fatty acids in microalgae oil a large percentage of the total fatty acid content, especially the docosahexaenoic acid (DHA) and eicosapentaenoic acid (EPA). Polyunsaturated fatty acid (PUFA) is a fatty acid that contains more than two double bonds in its backbone. According to the position of the first unsaturated bond, it is divided into ω3, ω6, ω7, and ω9, from which ω3 and ω6 play a vital role in body physiological function modulation. ω3 fatty acids have anti‐inflammatory, antithrombotic, antiarrhythmic, lowering blood lipid, and vasodilatation characteristics and are also essential for fetal and infant development, especially the brain and vision development. The main components of ω3 include alpha‐linolenic acid, 20‐carbon‐5‐enoic acid (EPA) along with 22‐carbon‐6‐enoic acid (DHA). The human body does not synthesize these three PUFAs and thus must be absorbed from the diet (Dyerberg & Jørgensen, 1982; Garg et al., 2006). Fishes or microalgae are the primary sources of ω3 PUFAs; microalgae‐derived PUFAs being cheaper than fishes. Additionally, compared to fish oil, ω3 PUFA extracts from microalgae are odorless and do not contain cholesterol (Mimouni et al., 2012).

**TABLE 3:** Percentage of PUFAs, DHA, and EPA in total fatty acids of different microalgae strains

Alga	Total PUFA %	EPA %	DHA %	References
*Aurantiochytrium*	60.73	0.75	49.79	Cui et al. (2019)
*Arcocellulus cornucervis*	–	14.8	2.26	Steinrücken et al. (2017)
*Attheya septentrionalis*	–	25.4	4.78	Steinrücken et al. (2017)
*Nitzschia laevis*	–	15.5	4.85	Steinrücken et al. (2017)
*Phaeodactylum tricornutum*	45.03	26.21	0.98	Qiao et al. (2016)
*Schizochytrium* sp.	62.98	0.86	52.28	Chen et al. (2016)
*Skeletonema menzelii*	49.07	12.08	3.84	Jiang et al. (2016)
*Phaeodactylum tricornutum*	–	20.3	2.91	Steinrücken et al. (2017)
*Thalassiosira hispida*	–	24.3	5.94	Steinrücken et al. (2017)

In the last few years, the study of microalgal PUFA has gathered interest increasingly. Jiang and Gao (2004) reported that reducing the growth temperature of a marine diatom, *Phaeodactylum tricornutum*, remarkably increased EPA and PUFAs yields. It indicated that *P. tricornutum* could be utilized as an aquacultural food organism as well as the candidate EPA source. Ryckebosch et al. (2014) assessed the nutrient contents of the total lipids isolated from the diverse PUFAs generated *via* microalgae. They used the microalgae, *Isochrysis* sp., *Nannochloropsis* sp., *Phaeodactylum* sp., *Pavlova* sp., and *Thalassiosira* sp. to produce ω3 PUFAs as a fish oil alternative in foods. *Skeletonema costatum*, *Chaetoceros calcitrans*, *Porphyridium cruentum*, as well as *Nannochloropsis* sp., showed high eicosapentaenoic acid (EPA) levels (Servel et al., 1994). High PUFA contents in these microalgae make them a good supplement for humans. In addition, Food and Drug Administration examined the safety of DHA and oils rich in AA after extraction from *Crypthecodinium cohnii* Javornicky single‐cell organism, a heterotrophic marine microalga, and recommended their use as supplements for infants (Martins et al., 2013). Because fish oil cannot satisfy the growing requirements of purified PUFA, large‐scale cultivation of microalgae is an excellent alternative. Different microalgal strains (149), in particular diatoms, were constructed to be the stock cultures, among which 20 were screened for growth rate and EPA/DHA contents, indicating their potential use in large‐scale production (Steinrücken et al., 2017).

### 3.1 Antioxidant activity

The recommended dietary intake of ω3 PUFAs for Chinese population is 200 mg~1 g (Punia et al., 2019). A high dose of polyunsaturated fatty acids may trigger oxidative stress, but a right dose has good antioxidant activity. Fu et al. (2016) studied the effect of DHA algae oil contained in edible oil on the antioxidant activity of SD rat and results showed that the proper supplementation of DHA can enhance the antioxidant capacity of the body. Zheng et al. (2017) also found that PUFA had an important influence in the peroxidation–antioxidant balance. They investigated the effect of DHA algae oil on promoting the body antioxidation and inhibiting the peroxidation reaction through measuring the contents of malondialdehyde, total antioxidant capacity, and catalase activity in cerebral cortex of SD rat. Results indicated that the moderate‐dose DHA alga oil played a positive role in enhancing free radical scavenging and reducing lipid peroxidation damage, but low and high doses showed adverse effects. Hence, the appropriate dietary intake of ω3 PUFAs from microalgae can improve the ability of scavenging oxygen free radicals and reduce peroxide damage of human body.

### 3.2 Antimicrobial activity

Microalgae has the potential to limit microbial infection in aquaculture and has great application prospect as a natural antibiotic. Now multiple studies have shown that short‐chain fatty acids and unsaturated chain fatty acids isolated from microalgae had antibacterial activity (Falaise et al., 2016; Senhorinho et al., 2015). Alsenani et al. (2020) identified the dominant compounds of three microalgae, *Isochrysis galbana*, *Scenedesmus* sp. and *Chlorella* sp. included, as DHA, EPA linoleic acid, and oleic acid, and the extracts of these fatty acids could inhibit the growth of gram‐positive bacteria.

### 3.3 Anti‐inflammatory activity

Long‐chain PUFA has therapeutic effects on diverse inflammatory diseases, like lupus, arthritis, or Alzheimer's disease (AD). Several animals and in vitro experiments supported the microalgal oil effect against inflammation. Nauroth et al. (2010) suggested that docosapentaenoic acid (DPA) inhibited the secretion of IL‐1β and TNF‐α by human monocytes in peripheral blood through in vitro LPS stimulation. Algal oil, which contained DPA (16% total fatty acids TFAs) as well as DHA (40%), significantly reduced inflammatory response in the model rats with foot edema compared to the control group. Banskota, Stefanova et al. (2013), Banskota, Roumiana et al. (2013) also suggested that a lipid extract isolated from *Nannochloropsis granulate* in RAW264.7 macrophage cells having activity against inflammation contained mono‐ and di‐galactosyldiacylglycerols. Dawczynski et al. (2018) were the first to explore the clinical parameters in patients with rheumatoid arthritis, who were allocated to consume microalgae oil from *Schizochytrium* sp, containing 2.1 g DHA/day. And results showed that supplemented DHA microalgae oil could ameliorate the state of an illness in patients along with rheumatoid arthritis, destroyed the balance of AA‐ and DHA‐derived lipid mediators, and presented an anti‐inflammatory/pro‐resolving state.

## 4 POLYSACCHARIDES (PS)

Several species of microalgae produced PS, sulfated exo‐polysaccharides in particular (Table 4), containing sugars like glucose, an aldose, galactose, and xylose (Raposo et al., 2013). PS in microalgae includes fucoidans, carrageenans, alginates, and exo‐polysaccharides. They were detected in microalgae as cell wall components, one part in cells peripheral to glycocalyx or one of the polymers outside the cells (or exo‐polysaccharides, EPS) (Table 4). *Spirulina* provides biological activities beneficial for tissue engineering. It could produce nanofibers and also contains various biomass contents to treat spinal cord damages or serve as the matrix in stem cell cultures (de Morais et al., 2010). PS isolated from *Spirulina platensis* is extensively utilized as a food additive or cosmetic colorant in Japan (Liu et al., 2016). *S. platensis* derived PS has been adopted as colorant for food items like ice sherbert, chewing gum, candy, popsicle, dairy product, soft drink, or jelly (Begum et al., 2016).

**TABLE 4:** Marine microalga species producing EPS

Microalgae	Group	Polysaccharide type	Main sugars	References
*Skeletonema costatum*	Diatoms	EPS	Glucose, aldose	Urbani et al. (2005)
*Cylindrotheca closterium*	Diatoms	EPS	Glucose, xylose	Staats et al. (1999)
*Phaeodactylum tricornutum*	Diatoms	EPS	Glucose, mannose	Guzmán‐Murillo et al. (2007); Chen et al. (2011)
*Haslea ostrearia*	Diatoms	sEPS		Rincé et al. (1999)
*Prorocentrum micans*	Pyrrophyta	EPS		Zhao et al. (2001)
*Trichodesmium*	Cyanophytes	EPS		Berman‐Frank et al. (2007)
*Aphanothece halophytica*	Cyanophytes	EPS	Glucose, fucose	Li et al. (2001)

Abbreviations: EPS, exo‐ or extracellular polysaccharide; sEPS, sulfate‐containing exopolysaccharide.

### 4.1 Antioxidant activity

Microalgae are photoautotrophs under high exposure to oxidative stress and radical stress and thus accumulate the efficient antioxidative PS that protect their cells from damage. Sun et al. used a hermetical microwave to degrade *Porphyridium cruentum* PS from 2,918 to 256.2, 60.66, or 6.55 kDa. High‐molecular‐weight (HMW) PS obtained from *P. cruentum* did not exhibit the antioxidation effect, whereas the low‐molecular‐weight (LMW) fragments, following degradation, showed inhibition against OS (Sun et al., 2009). In *Porphyridium* sp., sulfated exopolysaccharide sulfate showed an antioxidation effect on linoleic acid auto‐oxidation, while suppressed OS in 3T3 cells resulted from FeSO_4_. The PS from *Porphyridium* sp. inhibited oxidative damage depending on its dosage, which showed a positive correlation with sulfate levels in sEPS (Spitz et al., 2005). The EPS obtained from *Rhodella reticulata* exhibited an antioxidation effect, and crude sEPS showed an increased ability for free radical scavenging as well as superior antioxidation compared to PS‐modified samples (Chen et al., 2010a, 2010b). Also, Chen et al. (2010a), Chen et al. (2010b) found that extracellular crude PS from *Rhodella reticulata* could remove superoxide anion radicals and suppress linoleic acid auto‐oxidation depending on its dosage. Tannin‐Spitz et al. extracted PS from the red microalga, *Porphyridium* sp., and found that even after the degradation of PS, its components could scavenge free radicals (Tannin‐Spitz et al., 2005).

### 4.2 Antitumor activity and immunoregulation

The potential activity of PS is that it prevents the growth of cancer cells. EPS p‐KG03 containing high sulfate can be generated *via* red tide microalgae, *Gyrodinium impudicum* strain KG03. p‐KG03 showed immunostimulation activity and promoted the tumor‐killing effects of natural killer cells and macrophages as well as suppressed tumor cell growth in vivo (Yim et al., 2005). Geresh et al. (2002) suggested that “Oversulfated” EPS (with >20% sulfate level) with a high molecular weight at a concentration of 200 µg/ml could suppress the growth of 80% mammalian cells. Gardeva et al. (2009) demonstrated that sulfated polysaccharide from the cell wall of a red microalga, *Porphyridium cruentum* (Rhodophyta), showed an anticancer effect on Graffi myeloid tumor in hamsters in vitro and in vivo. After treatment with the sulfated polysaccharide, delayed tumor growth was observed with an extended median survival time by 10–16 days, thus suggesting that the *Porphyridium*‐derived EPS may serve as a potential candidate antitumor drug. Furthermore, Akao et al. found that PS isolated from *Spirulina platensis* could enhance the activity of natural killer cells in mice, thus play an essential role in antitumor (Akao et al., 2009). Park et al. (2011) reported the characterization of the water‐soluble polysaccharide from *Haematococcus lacustris* and determined its immune stimulation activity. The results indicated that PS had prominent immune‐stimulating activity and stimulated peritoneal macrophage RAW264.7 cells to increase the production of TNF‐α. Guzmán et al. (2003) studied the PS isolated from *Chlorella stigmatophora* and *Phaeodactylum tricornutum* and found that both the polysaccharides had different levels of anti‐inflammatory and immune modulatory activities.

### 4.3 Antiviral and antibacterial activities

Antiviral activity of the sulfated polysaccharides may be the most extensively investigated quality of marine microalgae. A marine microalga, *Cochlodinium polykrikoides*, generated sulfated polysaccharides outside the cell (Hasui et al., 1995). These PS constituted of mannose, galactose, glucose, and uronic acid, as well as the sulfate groups (S = 7%–8% w/w). They suppressed cytopathic impacts of influenza virus types A and B on Madin‐Darby canine kidney cells, as well as respiratory syncytial virus types A and B on HEp‐2 cells, and human immune deficiency virus type 1 on MT‐4 cells (Hasui et al., 1995). Radonic et al. suggested that *Arthrospira platensis* and *Porphyridium purpureum* released PS in the medium, which showed an antiviral effect on the Vaccinia virus and Ectromelia virus, in vitro or in vivo (Radonic et al., 2010). However, the mechanisms of this effect are not yet completely understood. Microalgae also showed antibacterial activity. Chen et al. (2005) found that the PS extracts from *Chlorella pacifica* and *Porphyridium cruentum* showed varied inhibitory activity against bacteria and fungi, where PS from *Chlorella pacifica* had strong antibacterial activity against *Rhizopus sinensis* and *Magnaporthe oryzae*.

### 4.4 Digestive performance

Dvir and colleagues reported that Sprague‐Dawley (SD) rats fed with PS from *Porphyridium* sp. caused a significant increase in feces bulk in animals, whereas decreased gastrointestinal transit time (Chayoth et al., 2000). Soluble EPS isolated from *Porphyridium* sp. may decrease the plasma and duodenal mucosal contents of blood lipid along with cholecystokinin, but increase fecal excretion of bile acid and neutral sterol. In addition, PS released by such red marine microalgae also induced morphological modifications within the colon and small intestine in SD rats. A notable decrease in goblet cell count in the mucosa layer was observed, along with increased fecal content viscosity. Moreover, the Jejunal tunica muscularis morphology showed enlargement. These morphological modifications could have taken place to overcome the nutrient and mineral malabsorption problems (de Jesus Raposo et al., 2016).

## 5 PIGMENTS

Natural pigments play a vital role during algae photosynthetic metabolism and pigmentation. Microalgae account for the primary photosynthesizers that generate several essential pigments, including chlorophyll, β‐carotene, phycobiliproteins, astaxanthin, and xanthophylls with applications in food, nutraceutical, pharmaceutical, aquaculture, and cosmetic industries (Table 5). Chlorophylls, carotenoids, together with phycobiliproteins, are the three primary classes of photosynthetic pigments of microalgae (Begum et al., 2016).

**TABLE 5:** Pigments from different microalgae

Microalgae	Group	Pigments	References
Green microalgae	Chlorophyta	Chlorophyll a and b, β‐carotene, prasinoxanthin, siphonaxanthin, astaxanthin	Begum et al. (2016); Van den Hoek et al. (1995)
Brown microalgae	Diatoms	Chlorophyll a and c, β‐carotene, fucoxanthin, diadinoxanthin	
Blue‐green microalgae	Cyanophytes	Chlorophyll a, xanthophylls, phycobiliproteins	
Dinoflagellates	Dinophyta	Chlorophyll a and c, β‐carotene, peridinin	

Most pigments are biologically active, with antiobesity, anti‐inflammatory, anticancer effects, mainly due to their potent antioxidant activity employed to protect the body from OS (Ciccone et al., 2013; Guedes et al., 2011). Phycobiliproteins are widely used in industrial and immunological research. As fluorescent markers, they are commonly used in immunoassays and as fluorescent dyes in molecular biology (Banskota, Roumiana et al., 2013; Banskota, Stefanova et al., 2013). Chlorophyll is a fat‐soluble pigment with a porphyrin ring, which accounts for 0.5%~1% of the dry weight of microalgae. Studies have found that chlorophylls have physiological effects like antimutagenesis and anticancer. Carotenoids, as essential nutrients, are mainly used in dietary supplements, fortified foods, food dyes, animal feeds, pharmaceuticals, and cosmetics (Vílchez et al., 2011). Carotenoids also show biological effects by affecting cell growth modulation, modulating gene level, or immune response (Kalra et al., 2020; Nascimento et al., 2020). Epidemiological studies showed that increased carotenoid consumption and tissue level were related to the decreased risks of malignancies and cardiovascular diseases (Rock, 1997). And it has been widely used as a sophisticated health product due to its activity to protect the heart and liver. Carotenoids have also been used in creams and lotions for sun protection as stabilizers and preservatives (Del Campo et al., 2000; Xhauflaire‐Uhoda et al., 2008). Humans and animals cannot synthesize carotenoids and rely on their diets for these essential nutrients. Thus, microalgae have been suggested to be a candidate source for carotenoids.

### 5.1 Chlorophylls

There are five types of chlorophyll in microalgae, including chlorophyll a, b, c, d, and e. Chlorophyll a is the primary photosynthetic pigment, abundant in Cyanobacteria and Rhodophyta. Chlorophyll b exists in Chlorophyta and Euglenophyta, while chlorophylls c, d, and e are found in the freshwater diatoms (Begum et al., 2016). *Spirulina* sp. is the most important chlorophyll a source, producing 2–3 times more chlorophyll than other plants. In addition, the chlorophyll in *Spirulina* contains porphyrin, similar to the heme in human and animals. It is a direct supplement of the heme in humans and animals, and thus, chlorophyll a is called “green blood.” Since *Spirulina* is rich in iron, the perfect combination of chlorophyll a and iron is the best treatment of iron deficiency anemia (Chamorro et al., 2002). Recently, chlorophyll has drawn significant attention for being used as a cancer preventative agent. Ferruzi and Blakeslee (2007) reported that the biological effects of the chlorophyll derivatives conformed to cancer prevention, including antioxidant and antimutagenic activities, mutagen trapping, xenobiotic metabolic regulation, and apoptosis induction.

### 5.2 Carotenoids

Carotenoids are the organic pigments *produced* by plants and algae, as well as several bacteria and fungi, but not synthesized in animals. Carotenoids from microalgae have different biological activities, especially related to human health. Carotenoids in microalgae mainly include astaxanthin, canthaxanthin, β‐carotene, lutein, and zeaxanthin. The main carotene‐rich microalgae are Chlorophyta, including *Chlorella*, *Scenedesmus*, *Chlamydomonas*, *Dunaliella*, and *Haematococcus*, as shown in Table 6 (Ibañez & Cifuentes, 2013; Mogedas et al., 2009). *Dunaliella salina* and *Dunaliella Bardawil* are the primary sources of β‐carotene, while astaxanthin and canthaxanthin are mainly found in *Haematococcus pluvialis* and *Chlorella*. In addition, *Scenedesmus almeriensis* and *D. bardawil* also contain β‐carotene while *H. pluvialis* and *Nannochloropsis* sp. have canthaxanthin.

**TABLE 6:** The main carotenoids derived from microalgae

Carotenoids	Microalgae
β‐Carotene	*Dunaliella salina*, *Dunaliella Bardawil*
Astaxanthin	*Haematococcus pluvialis*, *Chlorella vulgaris*
Canthaxanthin	*Haematococcus pluvialis*, *Chlorella vulgaris*
Violaxanthin	*Du‐ naliella tertiolecta*, *Chlorella ellipsoidea*
Lutein	*Nannochloropsis* sp., *Scenedesmus almeriensis*, *Chlorella protothecoides*

There are over 1,100 carotenoids in nature, among which β‐carotene is the most common (Yabuzaki, 2017). *Dunaliella salina* can accumulate large amounts of β‐carotene, accounting for 12% dry weight of cells. Compared with the chemosynthetic all‐trans structure, β‐carotene synthesized by *Dunaliella salina* has both all‐trans and 9‐cis structures and possesses higher bioavailability, antioxidant properties, and physiological functions. β‐Carotene from marine microalgae exhibits its antioxidative effects as a favorable scavenger of some chemical radicals, as well as the potent quenchers of singlet oxygen. β‐Carotene is not only known as a singlet oxygen quencher but also a potent scavenger of other ROS, including peroxyl radical (ROO•), hydroxyl radical (HO•), hypochlorous acid (HOCl), and anion peroxynitrite (ONOO⁻) (Rodrigues et al., 2012; Sachindra et al., 2007).

Astaxanthin is a kind of carotenoid not belonging to provitamin A. *Haematococcus pluvialis* is the primary source of astaxanthin. In addition, *Chlorella zofingiensis*, *Chlorococcum* sp., *Neochloris wimmeri*, *Catenella repens*, and *Coelastrella striolata* have also been reported to contain astaxanthin in lower content. The antioxidant performance of astaxanthin is better than that of β‐carotene, zeaxanthin, canthaxanthin, vitamin C, and vitamin E. It can relieve photooxidative stress and inhibit photosensitive action. In addition, astaxanthin proved to have a strong anticancer effect, which could reduce the number and size of liver cancer and lung tumor lesions, and it also has an obvious inhibitory effect on bladder cancer, oral cancer, and colon cancer cells (Sathasivam & Ki, 2018).

## 6 VITAMINS AND MINERALS

Microalgae are abundant sources for almost all important vitamins and minerals and rich in Cu, I, Fe, K, Zn, etc. (Christaki et al., 2011). Selenium (Se) is an indispensable element in the construction of glutathione peroxidase. Glutathione peroxidase participates in the antioxidant process of the human body, prevents the oxidation of unsaturated fatty acids, and avoids the production of some toxic metabolites, thereby reducing the risk of cancers and maintaining the homeostasis in the human body. In addition, Se also participates in the immune response by improving the proliferation and biological activity of B cells, improving the physiological function of T cells, and protecting the body's immune system by avoiding oxidative damage to cells.

The vitamins found in microalgae include vitamin B1, vitamin B2, vitamin B6, vitamin B12, vitamin C, vitamin E, niacin, biotin, and folic acid (Raposo & de Morais, 2015). Vitamin E, referred to as tocopherol, is a vital fat‐soluble antioxidant synthesized only by photosynthetic organisms. Microorganisms are an excellent source of natural V_E_. However, only photosynthetic algae, such as microalgae, can synthesize V_E,_ which mainly include *Spirulina platensis*, *Dunaliella tertiolecta*, *Synechocystis aquatilis*, *Nannochloro oculate*, *Tetraselmis suecica*, *Chlamydomonas*, *Ochromonas*, *Euglena gracilis*, etc. The V_E_ content in microalgae depends on many factors, such as genotype, growth stage, nutritional status, and light intensity (photosynthetic rate). Vitamin B_12_ has also been found in some macro‐ and microalgae. Edelmann et al. (2019) measured the vitamin B_12_ content in microalgae powders, whereas Chlorella sp. And N. gaditana powders solely contained active vitamin B_12_ up to 2.1 μg/g.

In addition, some vitamins have been shown to be produced by microalgae in recent years. The deficiency and insufficiency of vitamin D, belonging to a group of lipid soluble sterols, are a universal problem in the world. For example, more than one billion people are short of vitamin D_3_, especially around 13% European population on a yearly basis (Ljubic et al., 2020). Microalgae should be able to synthesize 7‐dehydrocholesterol in order to synthesize vitamin D_3_ by UVB exposure, if they use the same metabolic pathway as vertebrates. Ljubic et al. (2020) selected four microalgal species to produce vitamin D_3_ by exposure of artificial UVB, including *Chlorella minutissima*, *Nannochloropsis oceanica*, *Arthrospira maxima*, and *Rhodomonas salina*. Results showed exclusively *Nannochloropsis oceanica* could produce vitamin D_3_ with the yield up to 1 ± 0.3 g/g DM. In addition, increasing the dose of the UVB was able to significantly enhance the production of vitamin D_3_. Hence, *N. oceanica* exposed to artificial UVB could be used as a new natural source of vitamin D_3_. Recently, vitamin K_1_ has been identified as a potentially important nutrient to prevent the chronic diseases, especially some diseases associated with aging, such as osteoporosis and cardiovascular disease. Vitamin K_1_ is generally produced via chemical synthesis. However, currently, using microalgae to produce vitamin has a good application prospect. Tarento et al. (2018) screened seven different species of microalgae to produce vitamin K_1_. Results showed that *Anabaena cylindrica* was identified as the richest source of vitamin K_1_.

## 7 CONCLUSION

Previously, microalgae have attracted the interests of researchers because of their multiple sources and health benefits. This review highlights the bioactive substances and biological properties of marine microalgae and also illustrated the recent studies concerning biological activity and function. The bioactive substances include proteins, polysaccharides, pigments, and lipids. They demonstrate a series of physiological and pharmacological activities (e.g., nitrogen fixation, antitumor, antivirus, antioxidation, anti‐inflammatory, and antithrombotic) with applications in some medical and industrial fields. These bioactive substances from marine microalgae provide new and vast sources for the development of human health care and industrial production.

Although there are many advantages in using microalgae to produce high‐value products, its economic cost is too high due to a series of factors, such as low content of microalgae, low biomass, and much difficulty in harvesting, which greatly limited the utilization of microalgae resources. With the rapid development of synthetic biology, the use of this technology in microalgae artificially builds high‐value biosynthetic pathways of natural compounds to realize the goal of synthetic products (Saini et al., 2020). This is a faster and more economical way of producing. For example, in terms of increasing the yield of natural biosynthetic vitamin E, scholars mainly focus on increasing the yield of plant plants through metabolic engineering (Sproles et al., 2021).

The combination of technological and economic factors allows us to analyze the current structure of the microalgae industry and make some predictions. In the nutraceutics perspective, proteins and peptides based on microalgal have a wide range of applications that can produce high value‐added products and even use their properties to attract the cosmetic or pharmaceutical industries. The demand for DHA of consumers would greatly boost the production of PUFAs from microalgae. The market supply of PUFAs is still guaranteed by fish oil, but the PUFAs industry is likely to be one of the main drivers of microalgae to produce high value‐added products in the future. Microalgae polysaccharides are also attractive to the cosmetics industry because of their properties, for example, pushing cosmetic sectors toward other texturing agents. In addition, microalgae can be considered to be used for dyeing in the food industry because microalgae are rich in pigments. However, β‐carotene and astaxanthin possess diverse bioactive activities and will yield a variety of health benefits, with potential applications mostly in the cosmetics and pharmaceutical industries. In the future, microalgae are likely to be used not only in the food and feed industries, but also in the nutrition, health care, cosmetics, and pharmaceutical industries. Whether focusing on nutrition/health products or cosmetics/pharmaceuticals, the microalgae industry will be market‐oriented. In order to conform to this market trend, it has some challenges, such as it needs to select relevant varieties in the early stage of cultivation, and producers will need to start from product development. Given the diversity of microalgae, this may not be an easy choice. Moreover, active substances of microalgae are extracted, produced and priced according to their different positive effects, and its industrialization development technology also needs further in‐depth study.

## CONFLICT OF INTEREST

None.

## AUTHOR CONTRIBUTIONS

**Jinhong Wu:** Conceptualization (lead); Data curation (equal); Formal analysis (equal); Investigation (lead); Methodology (equal); Writing‐original draft (lead). **Xinzhe Gu:** Conceptualization (equal); Data curation (equal); Formal analysis (equal); Investigation (equal); Methodology (equal); Writing‐original draft (lead); Writing‐review & editing (lead). **Danlu Yang:** Data curation (equal); Formal analysis (equal); Investigation (equal). **Shannan Xu:** Formal analysis (equal); Project administration (lead); Resources (equal); Supervision (lead). **Shaoyun Wang:** Investigation (equal); Methodology (equal); Project administration (equal); Supervision (equal). **Xu Chen:** Data curation (equal); Formal analysis (equal); Project administration (equal). **Zhengwu Wang:** Investigation (equal); Methodology (equal); Project administration (equal); Resources (equal); Supervision (equal).

## Data Availability

The data that support the findings of this study are available from the corresponding author upon reasonable request.
